# “I did not know it was so important to take it the whole time” − self-reported barriers to medical treatment among individuals with asthma

**DOI:** 10.1186/s12890-019-0934-3

**Published:** 2019-09-18

**Authors:** Tove Hedenrud, Annika Jakobsson, Hanan El Malla, Helle Håkonsen

**Affiliations:** 1 Department of Public Health and Community Medicine, University of Gothenburg, P.O. Box 453, 405 30 Gothenburg, Sweden; 2 Department of Social Work, University of Gothenburg, P.O. Box 720, 405 30 Gothenburg, Sweden

**Keywords:** Barriers, Medical treatment, Asthma, Medicines, AAAQ, Qualitative

## Abstract

**Background:**

Asthma is an extensive public health problem and inadequate disease control is not uncommon. Individuals’ self-perceived barriers to medical treatment for the entire treatment chain (from seeking care for symptoms to using a medicine) have seldom been studied for chronic diseases such as asthma. The aim of this study was to explore self-perceived barriers to medical treatment among individuals with asthma within the framework of AAAQ (availability, accessibility, acceptability and quality).

**Methods:**

Individuals with asthma visiting the asthma nurse at a primary health care centre, and who currently had a prescription for anti-asthmatic medicines, were informed about the study. The nurse asked the persons for their consent to be contacted by an interviewer. The interview guide was constructed from the elements of AAAQ exploring self-perceived barriers to asthma treatment. Interviews were conducted in Swedish, English, Arabic and Persian. They were transcribed verbatim and a manifest content analysis was conducted.

**Results:**

Fourteen interviews were conducted. There was a large variation in both age and reported number of years with asthma. Self-perceived barriers to asthma treatment were experienced throughout the whole treatment chain. Barriers that emerged were health care accessibility, perceived quality of care, beliefs about medicines, life circumstances, knowledge gap about asthma and medicines, practical obstacles to using medicines, and experiences with treatment. The self-perceived barriers cover all four elements of AAAQ, but there are also some barriers that go beyond those elements (life circumstances and practical obstacles to using medicines).

**Conclusions:**

Self-perceived barriers among individuals with asthma cover the whole treatment chain. We want to highlight the inadequate information/education of patients leading to knowledge gaps about both disease and the effect of medicines, and also the perceived unsatisfactory treatment at the PHCC, which could partly be counteracted if patients know what to expect from health care visits.

## Introduction

Medical treatment of chronic diseases is far from optimal, leading to consequences for both the individual and society, one example being asthma [1]. Asthma is an extensive public health problem, both in Sweden and world-wide [2]. The prevalence is about 10% in Sweden [3]. The disease requires prophylactic and/or acute treatment [4], and a large degree of self-management is involved. Previous research has reported that individuals with asthma have inadequate disease control [5, 6] and a lower quality of life compared to people without asthma [7]. Consequences for the society are increased use of health care services and indirect costs associated with unproductivity [8].

Individuals’ self-perceived barriers to medical treatment for the entire treatment chain (from seeking care for symptoms to using a medicine) have seldom been studied for chronic diseases such as asthma. Instead, barriers to treatment of chronic diseases have previously been studied from a health care perspective [9, 10], and some qualitative research has addressed barriers and facilitators to access to and utilization of health care services [11–13]. The identified barriers are both general (low income, communication problems, lack of knowledge about the health care system, disagreeable treatment by clinic staff) [9, 11], and specific to a disease (underdiagnosing of men, unawareness of benefits of treatment, stigmatization, values and attitudes of health care provider) [9, 10, 12]. Obstacles to adherence have also been discussed, both in general, and specifically for asthma treatment, for instance [14–16]. Still, there is a need for a more comprehensive picture of all barriers perceived by those in need of medical treatment that will provide tools for researchers, health care providers and policy makers to improve the management of chronic disease, and in this case, asthma specifically.

The right to the highest attainable standard of health (from here on, the Right to Health) is inscribed in many human rights treaties. The Right to Health encompasses the elements of availability, accessibility, acceptability and quality (AAAQ) [17], which may be used as a framework to identify barriers over the entire treatment chain. Availability entails ensuring functioning health services in sufficient quantity, whereas accessibility entails ensuring that health services are non-discriminatory and physically and economically accessible, and that information is accessible. Acceptability implies that health services must be respectful of medical ethics, culturally appropriate, and gender and age sensitive, and that medical treatment must be explained in an understandable manner. Finally, quality requires that health facilities and medicines are scientifically and medically appropriate and of good quality. Schierenbeck et al. used the AAAQ as a framework in their analysis of mental health care in South Africa and found barriers pertaining to all four elements [10].

The aim of this study is to explore self-perceived barriers to medical treatment among individuals with asthma within the framework of AAAQ.

## Methods

Since there is little knowledge about self-perceived barriers to treatment, we chose to explore this area with a qualitative study. The qualitative approach enables a description of individual experiences of medical treatment and perceived barriers from the participants’ unique perspectives.

### Setting

In Sweden, health care is primarily financed through taxes, and primary care is responsible for basic medical treatment and is the first level of care for the citizens. The out-of-pocket copayment for a visit to a primary care physician varies between €10 and €30 (US$12–36). The maximum annual cost for health care visits is €112 (US$135). Sweden has about 1300 pharmacies (about 14 pharmacies per 100,000 inhabitants) where prescription drugs are dispensed [18]. The maximum annual copayment is approximately €225/year (US$270) for medicines included in the reimbursement system. In the western part of Sweden (Region Västra Götaland), all primary health care centres (PHCC) have an asthma/chronic obstructive pulmonary disease (COPD) nurse employed [19].

### Participants

Participants were recruited through two primary health care centres (PHCCs) in the city of Gothenburg (about 550,000 inhabitants; 23% born abroad) by an asthma nurse who worked in both places. One PHCC was situated in the city centre, the other in an area with a large proportion of foreign-born residents. The data collection started in September 2016 and continued until June 2017, 3 days per week. To gather data as rich and substantial as possible, efforts were made to include both men and women, and individuals having experienced asthma for a varying number of years. Further, to facilitate and increase participation, data collections from study participants were conducted in Swedish and English, and in two of the other most common languages among inhabitants in Gothenburg (Arabic and Persian). Two interviewers were involved in the project: one conducted interviews in Swedish and Persian, and the other in English and Arabic (while also being fluent in Swedish). The most experienced qualitative researcher (AJ) performed a training session in interview skills. The Persian-speaking interviewer has a background in nutrition and as a pharmacy technician. The Arabic-speaking interviewer (HEM, PhD in medicine) has a good understanding of regional differences in the Arabic language and a professional background in psychology. One interview was conducted by the first author, who is a pharmacist (TH).

All individuals with asthma visiting the asthma nurse at one of the PHCCs, and who currently had a prescription for anti-asthmatic medicines, were informed about the study. Both verbal and written information (available in all four languages) was provided. It was emphasized that participation was voluntary and that lack of interest in participating would not affect the care provided at the PHCC. Individuals who could not be interviewed in any of the four languages were not asked to participate. The same applied to individuals with COPD or impaired cognitive ability. The nurse asked the persons for their consent to be contacted by an interviewer, their preferred means of contact (e-mail or telephone), and their preferred language. This information was forwarded to the project coordinator. Ethical approval was received by the Regional ethics board in Gothenburg (Dnr: 192–16).

### Interviews

We elaborated procedures for all steps of the process that each interviewer used in their contacts with potential participants. The preferred interviewer contacted the potential participant and they decided a time to meet if the person was still interested in being part of the project. The interviews were conducted either in an interview room at the Section of Social Medicine and Epidemiology (University of Gothenburg), or in a meeting room at a public or university library. At the meeting, the purpose of the study was explained to the participants as was the need to audio-record the interview. The interviewer asked whether the person had read the information leaflet and whether there were any questions. Finally, the person was asked to sign the consent form.

The face-to-face interview started with background questions on sex, age, number of years of schooling, and country of origin. The interview guide was constructed from the elements of AAAQ [17]. The interview continued by asking the participants to tell about their experiences with and perceptions of their disease, and to give their opinions on the asthma treatment they received (Appendix). The interviewer had a folder with photos of different inhalers (controller and rescue medicines) where the participant could point out which ones were used. Then the key theme was introduced by asking whether they at any time had experienced any barriers to treating their asthma, which was expected to be a starting point for individuals to tell about their self-perceived barriers. In most cases this was perceived to concern administration of medicine or issues related to adherence. Hence, the prepared probing questions were used to cover all elements of AAAQ. The interviews had a life-course perspective, that is, the barriers could have been experienced at any time. The first two were pilot interviews to test the interview guide. It was subsequently decided that these interviews should be included, since the interview guide was not revised after the pilot interviews. After the interview, the participant was asked to fill out the Reactions to Research Participation Questionnaire (RRPQ-P) [20] in the preferred language. The RRPQ-P is a brief measure of how the research participation was experienced. Finally, the participants were given contact information for those responsible for the study.

The interviews varied in length between 15 and 88 min (mean: 40 min), and they were all audio-recorded and transcribed verbatim. Interviews conducted in Persian and Arabic were both transcribed and then translated into Swedish by the person who had conducted the interview. Interviews in English were not translated into Swedish.

### Analysis

Before the inclusion of participants started, we decided that at least 12 interviews should be conducted, based on results from a previous study on saturation of qualitative data collections [21]. After 12 interviews, another two interviews were conducted, and since only a few new codes were identified, the data collection was ended. NVivo 11 software was used for data management (QSR International Pty Ltd., Melbourne, Australia).

The analysis was performed by two of the authors with different professional backgrounds: TH is an Associate professor and a pharmacist and AJ is a Doctor of medicine and a registered nurse. A manifest content analysis was performed, inspired by Graneheim and Lundman [22], which is an analysis of what the text says without any interpretation by the researcher. Hence, the analysis describes the visible, obvious components of the text and avoids interpreting the participants’ statements. First, each interview was read through to gain a common understanding of the entirety. Then, the interview was read line by line to identify meaning units. A meaning unit could vary from a few words to a sentence to a paragraph. For two interviews, meaning units were condensed before being assigned a code. This was done to compare how codes were assigned by the two researchers performing the analysis. Consensus was obtained for all codes. All coauthors sorted the resulting codes into categories, and these categories were discussed at a seminar with other researchers. The categories were revised somewhat after the seminar. See Table 1 for examples of meaning units, codes, subcategories and categories. Throughout the process of analysis, any disagreements were resolved through discussions.

**Table 1:** Examples of meaning units, codes, subcategories and categories

Meaning unit	Code	Subcategory	Category
Not all the pharmacies have the medicines I’m looking for	Medicine not in stock	Accessibility of primary health care centre (PHCC) and pharmacy	Health care accessibility
they contain cortisone. And you’ve always heard that it’s dangerous. So you do not want to get it in you, I think.	Cortisone is dangerous		Beliefs about medicines
Going away for a few months, you have to fill half the bag with only medicine.	Practical problems when traveling	Medicines, travel and sports	Practical obstacles to using medicines

## Results

Fourteen interviews were conducted (8 women, 6 men). There was a large variation in both age (18–72 years) and reported number of time with asthma (from 2 months to 43 years). All but one participant had been diagnosed with asthma in Sweden. Number of years of schooling varied between null and 18 years. Eleven individuals had 12 years or more of schooling. Nine interviews were conducted in Swedish, three interviews in Persian and one each in English and Arabic. Among migrants, time in Sweden varied between 2 and 21 years.

Seven categories emerged from the manifest content analysis (Table 2), describing the self-perceived barriers to asthma treatment. *Health care accessibility* comprised two subcategories: accessibility of the PHCC and pharmacy, and finances. The category *perceived quality of care* also had two subcategories: treatment at the PHCC and inadequate diagnostics. *Beliefs about medicines* encompassed negative perceptions of having to take medicines. *Life circumstances* included social and health issues that impact on treatment. In the category *knowledge gap about asthma and medicines*, codes comprised perceived lack of knowledge that affects daily use of medicines. *Practical obstacles to using medicines* had two subcategories: the first being medicines, travel and sports, and the second, forgetfulness. The final category, *experiences with treatment*, covered experiences of inadequate effects and adverse effects of the medicine.

**Table 2:** Resulting categories and subcategories describing self-perceived barriers to treatment

Categories	Subcategories
Health care accessibility	• Accessibility of primary health care centre (PHCC) and pharmacy • Finances
Perceived quality of care	• Treatment at PHCC • Inadequate diagnostics
Beliefs about medicines	
Life circumstances	
Knowledge gap about asthma and medicines	
Practical obstacles to using medicines	• Medicines, travel and sports • Forgetfulness
Experiences with treatment	

### Health care accessibility

This category covered how the participants perceived the possibility of contacting the PHCC and getting an appointment, whether they could get their medicine at the pharmacy, and the costs for care and medicines.

Concerning the accessibility of the PHCC, the participants had experienced problems with the telephone system of the PHCC (not getting through), opening hours that were not compatible with their own working hours, difficulties in getting an appointment with a doctor to renew prescriptions and low accessibility of the asthma nurse (worked 1–2- days at each PHCC).

*“So then you get stuck in a queue, maybe wait one to two weeks, or that all doctors should come home from their holidays, to get to one’s doctor, to get the prescription…”. (interview 2)*

*“My problem with this health care centre is that you can only meet the asthma nurse once a week”. (interview 11)*

Several participants mentioned that their medicine was never in stock at the pharmacy but always had to be ordered, which took a few days. How this delay was experienced varied among the participants, and for some it was perceived as a barrier to treatment.

Financial aspects were addressed in all interviews, but it was not experienced as a barrier by all participants, since their financial circumstances differed (income, health insurance). Several participants stated that medicines were costly but that they prioritized the cost, since they needed their treatment. Economic barriers mentioned were lost working hours due to doctors’ visits, the cost of medicines and the high cost of seeking emergency care.

*“I can, of course, go to the emergency ward, but it is more expensive and it is difficult for pensioners”. (interview 14)*

The high cost of getting certificates for medicines to take abroad was mentioned, and also the cost of purchasing medicines abroad.

### Perceived quality of care

The experiences of how individuals were received and treated at the PHCC were included in this category, together with delays in getting the right diagnosis.

Participants had experienced delays in obtaining a diagnosis of asthma, despite repeated care seeking.

*“ … I have been contacting a doctor a number of times because it is an incredible amount of cough and phlegm. But it has not been taken seriously…”. (interview 1)*

A foreign-born participant, who did not speak Swedish, had met different doctors (an Arab and a Swede) at two different PHCC visits and was confused about the diagnosis as the doctors had used different expressions about the disease (“asthma” and “rabou”). Hence, he believed he had two diagnoses.

Foreign-born participants had experienced that the doctors had inadequate knowledge of English but also expressed a lack of confidence in Swedish doctors.

*“And also the fact that doctors not all the time are able to express themselves in English”. (interview 4)*

*“I have more confidence in Iranian doctors. They dare more to provide diagnosis. No, I do not feel comfortable with Swedish doctors at the health care centre”. (interview 9).*

They experienced that their situation had not been taken seriously at the PHCC, they did not receive the medicine they wanted, and they perceived a lack of information. Lack of information was mostly expressed in general terms, but an example was also given of how information on how to use an inhaler was first provided after 10 years of medicine use.

### Beliefs about medicines

Participants expressed a reluctance to take medicines, especially if they contained cortisone. Because of this, one of them had stopped taking the medicine, and the asthma had deteriorated.

*“I do not like so much taking them, so I avoid them sometimes, because I... It does not feel like you should take such substances. So then I've stopped taking them sometimes if I feel fine”. (interview 5)*

### Life circumstances

Acute illness and social circumstances impacted on the energy to continue with the medical treatment of asthma.

*“I got an inhaler that I used very irregularly. One day I used it and the next day I did not to be honest. I was very careless and did not take it seriously. At the time my life problems were extremely demanding and I could not think of my asthma”. (interview 11)*

### Knowledge gap about asthma and medicines

A lack of knowledge about the necessity of continuous medical treatment affected the use of asthma medicines. The knowledge gap in combination with a lack of symptoms led to nonadherence.

*“I thought that this was something you would have only half a year, then it was gone. So, I avoided the medicines for a year maybe. I did not know it was so important to take it the whole time”. (interview 10)*

Other aspects that were mentioned, although not expressed as barriers, were the need for more information when receiving the diagnosis, and more knowledge about self-management of impaired asthma status.

### Practical obstacles to using medicines

Participants raised the impracticalities of using medicines in relation to travel and sports. The inconvenience of always having to carry asthma medicine everywhere was mentioned, as was the risk of running out of medicine while travelling. In some cases a certificate is needed, and a long stay outside the country means having to take a lot of packages.

*“Going away for a few months, you have to fill half the bag with only medicine”. (interview 5)*

Forgetfulness was addressed in most interviews. Participants admitted forgetting to take their medicine, to renew a prescription or to schedule a new appointment with the doctor. Several described their daily routine for taking their medicine and said that if this changed they also forgot to take it.

*“When I’m not in my routine, I would say. Because usually I have a daily routine: when I wake up, I take it and then I do some other stuff, and when that, something is different, then I forget about it”. (interview 4)*

### Experiences with treatment

This category covered experiences of inadequate effects and adverse effects of the medicine.

One participant explained his experience of inadequate effect of medicines as follows:

*“…it does not really help with the medicine I have, even though we have changed and tried different ones”. (interview 12)*

Other aspects mentioned were experienced adverse effects, perceived variation in effect of medicine over 1 year and difficulty of knowing whether the medicine enters the lungs.

*“I am uncertain if I get, if I manage to inhale enough of the powder, if I get it down properly. So that, in that way I think it is better with puffs, that it ejects a puff by itself”. (interview 1)*

It was also suggested that if the medicine had a taste, it would be easier to feel sure that they had actually inhaled the medicine correctly.

Results from the RRPQ showed that all interviewees agreed or strongly agreed that they participated voluntarily (“It was my choice if I was in the study”). All but one interviewee agreed/strongly agreed to having received accurate information about the study beforehand.

## Discussion

The results from this study of self-perceived barriers to medical treatment among individuals with asthma show that barriers are experienced throughout the whole treatment chain, from accessibility to the PHCC to forgetfulness and experiences with treatment. The self-perceived barriers cover all four elements of AAAQ, but there are also some barriers that go beyond those elements (life circumstances and practical obstacles to using medicines).

Lack of *availability* is part of the category health care accessibility, where the participants highlighted difficulties in getting appointments at the PHCC, or wait times for medicines to be available at the pharmacy. This confirms results from a previous study on barriers to asthma guidelines [23]. These barriers are issues for policy makers to address. Lack of appointments in primary care could entail a need for emergency care [24], which is more costly for society as well as the individual and could become a barrier for low-income citizens. A European survey found that two-thirds of unmet need for health care was due to waiting lists and appointment availability [25]. Concerning available medicines at the pharmacy, all Swedish pharmacies are obliged to deliver a requested prescription drug within 24 h. About 95% of Swedish pharmacy customers have their medicines dispensed at the first pharmacy visit [18], but with a total of 86 million prescriptions dispensed during 2017, there is still a large number of customers who leave the pharmacy without their medicine.

Inadequate *accessibility* was expressed in terms of opening hours at the PHCC, or not being able to contact the PHCC. This is experienced in other countries too and is perceived as unsatisfactory [11, 24–26]. However, accessibility may be difficult to compare between countries, due to different health care systems and pharmacy regulations. Regarding financial accessibility, the cost of medicines was not a big issue among our participants in contrast to previous results [13, 23]. This is largely because of the Swedish Pharmaceutical Benefit Scheme, which aims to protect all citizens from high health care costs. Further, people living on social security are exempted from copayment. In a population survey performed by the Public Health Agency of Sweden in 2014, 6% reported that they did not purchase their prescribed medicine at the pharmacy of whom 20% stated financial reasons [27]. This result is confirmed in an international comparison [28]. The importance of finance as a barrier to treatment among individuals with asthma in Sweden will be elucidated in a subsequent quantitative study. It is possible that the costs of medicines are experienced as a larger problem on a group level among individuals with asthma.

There was a lack of access to information expressed through the category knowledge gap, which concerned a lack of understanding about the necessity of continuous medical treatment, even during periods when they felt the disease was under control. This result supports previous research showing that non-use of inhaled corticosteroids was related to a belief that the medicine was unnecessary during asymptomatic periods [29]. In a French study, one-third of the participants interrupted their use of inhaled corticosteroids when feeling better [30]. Overcoming the knowledge gap is an educational task, primarily for physicians and asthma nurses, which needs improvement, repetition and follow-up. The physician is especially important as shown by previous research, reporting that the physician is perceived as the most reliable source of information [23], and is expected to provide basic information [13]. Information may also be reinforced at the pharmacy when asthma medicines are purchased. However, a persistent challenge is that patients tend to forget a considerable amount of the information given by health care personnel [31].

The element of *acceptability* included treatment at the PHCC, beliefs about medicines, and to some extent, experiences with treatment. Concerning treatment at the PHCC, a lack of information was highlighted in several interviews, even though it was not always mentioned as a barrier to treatment. More information in general, particularly when the diagnosis is new, was requested, but also more specific requirements were mentioned. The perceived lack of information is partly dependent on expectations, which may be more or less realistic on an individual level. The participants were recruited through PHCCs and may therefore be more prone to mention a perceived lack of information from health care personnel. However, pharmacy staff also has a responsibility to inform their customers about, for example, the administration of medicines. Difficulties with inhalers is a very common problem [13, 23, 32]. The perceived uncertainty whether the medicine enters the lungs (experiences with treatment) is also a question of adequate information about medicine. To start with, the physician must explain clearly what asthma is and how the medicine works to improve health. One should also be able to expect that the asthma nurse and/or the pharmacist would make certain that the individual could inhale the asthma medicine properly. Individual forgetfulness is also an aspect; an instruction that was received years ago may no longer be recalled.

Other aspects of treatment at the PHCC perceived as barriers are related to culture, where foreign-born individuals highlighted aspects concerning Swedish doctors, their communication and practices. Not being prescribed a requested medicine, or experiencing not being taken seriously, affected the treatment satisfaction. Another Swedish study reported that foreign-born individuals described a lack of trust in their Swedish doctors and that they did not appreciate the less paternalistic approach of doctors in this country [33]. A recent Norwegian study reported similar results, where the doctors’ treatment and prescribing practices were mentioned as barriers by interviewed migrants [11]. Our results also confirm the perceptions of care professionals across Europe, who reported cultural differences and lack of familiarity with the health care system as frequent problems when providing care for migrants [34]. It is clear that information about a national health care system should not only be a health care service guide. It should also convey what to expect at a physician visit to prevent false expectations about practices and prescription habits. Further, health care staff need continuous education and time for reflection on transcultural patient encounters [35].

The *quality* elements in our results are the subcategories inadequate diagnostics and experiences with treatment, particularly the aspects of adverse effects, and inadequate effect of medicines. Experienced adverse effects were mentioned as a barrier, but overall considered endurable. Concerns about the potentially negative effects of medicines, and their effect on adherence to treatment, have been reported in numerous studies [13, 23, 36–38]**.** The participants who reported a delay in getting a diagnosis of asthma mentioned having airway problems for several years before they were diagnosed. Low adherence to diagnostic procedures for asthma and COPD has been reported in a Swedish study of primary care [39], which indicates an underdiagnosing of both diseases.

Concerning practical obstacles, forgetfulness is a well-known barrier to adherence to medicines or to doctors’ appointments [40]. Routines were reported to be helpful for our participants in remembering to take the medicine. Previous research showed that a mobile phone short message service might enhance adherence to medicine [41].

Although access to care should be based on need [42], there are numerous examples showing that this is not achieved. An evaluation of the free choice of primary health care centre reform (implemented in Sweden in 2010) found that individuals with higher needs of care and lower socioeconomic position have not benefited from the reform [43]. Further, there is a socioeconomic gradient in medicine utilization in Sweden, for example, highly-educated women use more medicines in general, and more expensive medicines, than women with lower education [44]. A review of social disadvantage and asthma control in children found that the presence of factors such as low socioeconomic status, psychosocial stress and minority affiliation were linked to worsened asthma outcomes [45]. Further, another review found that ethnic minorities to a lesser extent received prophylactic asthma treatment, which led to an increase in hospitalizations [46]. The barriers identified in this study will be transformed into quantitative variables to measure the frequency of barriers among people with asthma. More importantly, it will be investigated whether barriers differ between people in relation to social factors such as education or the need of an interpreter at medical consultations. The presence of barriers will also be analysed in relation to asthma status and health outcomes.

Regarding ethics, the asthma nurse asked the participants about their interest in participating, which could entail a risk of individuals feeling pressured to join the project. However, it was not until they eventually met the interviewer that the agreement form was signed. Further, as shown by the RRPQ, the participants reported that participation in the study was their own choice. Another question of particular importance concerned correct information, since our participants had different backgrounds and spoke different languages. All but one (Swedish-speaking) participant agreed to having received correct information beforehand; hence, the translations of the information material seemed understandable.

### Methodological aspects

According to the method used [22], it is desirable to identify themes that are at the latent level. In this study, the categories were quite disparate and we chose not to force the categories into themes. Accordingly, we preserved closeness to the data. In our analysis, seven categories emerged. These refer to the descriptive level and constitute an expression of the manifested content of the analysed interviews.

The number of interviews was considered sufficient, and the results should cover the most important barriers perceived by individuals with asthma but we do not claim saturation of data. In general, as found by Francis et al. [21], more interviews would not increase the number of codes substantially. The characteristics of participants also differed in terms of sex, age and number of years with asthma.

A major strength of this study is the interviews with non-Swedish speaking individuals, which enriched the data and enhanced the quality of the project. Further, it is a question of fairness that everyone should have the possibility to contribute to research. However, it also entails some challenges, with several steps of translations, which could impact negatively on the quality of the results [47, 48]. Our interviewers, who were familiar with medical terminology, have been involved in the whole data collection process; one is a co-author and the other was active at the research seminar and has read and concurs with the results. The research process has been systematic and transparent, and any ambiguities have been discussed among the researchers. Another strength is the involvement of researchers with different professional backgrounds, first in the analysis and then at the research seminar, where both researchers and practitioners participated.

## Conclusion

Self-perceived barriers among individuals with asthma cover the whole treatment chain, from accessibility to the PHCC to forgetfulness and experiences with treatment. We want to highlight the inadequate information/education of patients leading to knowledge gaps about both disease and the effect of medicines, and also the perceived unsatisfactory treatment at the PHCC, which could partly be counteracted if patients know what to expect from health care visits.

## Data Availability

The data used in this study are available from the corresponding author on reasonable request.

## Appendix

### Interview guide

*Introductory themes*

- How long have you had problems with asthma?
- What sort of medications do you take for your asthma? A) Open question; B) Show inhalator guide
- What do you think about taking medication for asthma (medication prescribed by a doctor)?
- In which situations do you forget to take your asthma medication?
- In which situations do you skip a dose or take more than the doctor has prescribed?
- What can you tell me about the time between your first asthma symptoms and when you got a medical treatment that worked (or until today)?
- Do you experience any barriers to treating your asthma?
  - What are they?
  - Specific questions – see below.

*Exploratory questions (can be used if the interview subject gives limited information)*

1. When you sought treatment for your asthma, how did you experience that the medical staff treated you? (Communication/information/language/staff competence)
2. Do you experience that you have enough knowledge to take your asthma medication?
  - What knowledge do you have? How did you get that knowledge?
  - Is there anything that you need to know, but do not know?
  - How/in what ways/why do you find that your present knowledge is sufficient/insufficient?
3. Do you experience any barriers in getting to and from your medical centre or pharmacy?

- What are the obstacles?
- Why/in what ways are these obstacles?

4) Do you experience any financial barriers to treating your asthma?
  - What financial obstacles?
  - How/in what ways do you experience these as obstacles?
5) Do you experience that waiting times are barriers in being able to treat your asthma?

- Which waiting times?
- How/in what ways do you experience these as obstacles?

6) Do you experience that side effects are barriers in being able to treat your asthma?
  - Which side effects?
  - How/in what ways do you experience these as obstacles?
7) How do you experience that your family/friends relate to your medications?
8) Do you have any other illnesses or conditions? What do you feel about these in relation to your asthma?

## Abbreviations

AAAQ: Availability, Accessibility, Acceptability, Quality
COPD: Chronic Obstructive Pulmonary Disease
PHCC: Primary Health Care Centre
RRPQ-P: Reactions to Research Participation Questionnaire
